# rESWT promoted angiogenesis via Bach1/Wnt/β-catenin signaling pathway

**DOI:** 10.1038/s41598-024-62582-2

**Published:** 2024-05-22

**Authors:** Fan Yang, Juan Guo, Nan Kang, Xiaotong Yu, Yuewen Ma

**Affiliations:** 1 Department of Rehabilitation Medicine, Suzhou Municipal Hospital, Suzhou, 215002 China; 2 Department of Rehabilitation Medicine, The First Affiliated Hospital of China Medical University, Shenyang, 110001 China; 3 Institute of Meta-Synthesis Medicine, Beijing, 100097 China

**Keywords:** Radial extracorporeal shock wave therapy, Angiogenesis, Transcriptional regulator Bach1, Wnt/β-catenin signaling pathway, Vascular endothelial growth factor, Cellular neuroscience, Stroke, Translational research, Molecular medicine, Neurological disorders

## Abstract

Previous reports have established that rESWT fosters angiogenesis, yet the mechanism by which rESWT promotes cerebral angiogenesis remains elusive. rESWT stimulated HUVECs proliferation as evidenced by the CCK-8 test, with an optimal dosage of 2.0 Bar, 200 impulses, and 2 Hz. The tube formation assay of HUVECs revealed that tube formation peaked at 36 h post-rESWT treatment, concurrent with the lowest expression level of Bach1, as detected by both Western blot and immunofluorescence. The expression level of Wnt3a, β-catenin, and VEGF also peaked at 36 h. A Bach1 overexpression plasmid was transfected into HUVECs, resulting in a decreased expression level of Wnt3a, β-catenin, and VEGF. Upon treatment with rESWT, the down-regulation of Wnt3a, β-catenin, and VEGF expression in the transfected cells was reversed. The Wnt/β-catenin inhibitor DKK-1 was utilized to suppress Wnt3a and β-catenin expression, which led to a concurrent decrease in VEGF expression. However, rESWT treatment could restore the expression of these three proteins, even in the presence of DKK-1. Moreover, in the established OGD model, it was observed that rESWT could inhibit the overexpression of Bach1 and enhance VEGF and VEGFR-2 expression under the OGD environment.

## Introduction

As a form of physical therapy, radial extracorporeal shock wave therapy (rESWT) demonstrates exceptional efficacy. The characteristics of rESWT include non-invasiveness, and relatively simple operation. In recent years, rESWT has not only been commonly used in treating bone and muscle diseases, but it has also demonstrated remarkable effects in treating wound, urology disease, peripheral nerve injuries, cellulite, among others^1–6^. Recent animal and clinical studies have revealed that rESWT exerts a significant therapeutic effect by promoting angiogenesis at the injury sites^7–10^. Reports suggest that rESWT improved blood flow in rats with diabetic foot ulcers^11^. Previous studies have shown that the application of rESWT to ischemic stroke rats leads to better recovery of cerebral blood flow in the infarction volume by increasing the expression of vascular endothelial growth factor (VEGF)^12^. rESWT has also been shown to exert significant therapeutic effect in male erectile disorders, facilitating blood flow in the cavernous tissue^13^.

Angiogenesis, the process of new blood vessel formation in endothelial cells, is regulated by several signaling pathways and pro-angiogenic factors. These include the Wnt/β-catenin signaling pathway and its downstream target gene, VEGF^14,15^. VEGF is a crucial factor contributing to angiogenesis, playing an essential role in both physiological and pathological angiogenesis^16^. As a newly discovered factor related to angiogenesis, BTB (broad complex, tramtrack, bric-a-brac) and CNC (cap'n'collar) homology 1 (Bach1) serves as critical transcriptional regulators in mammals and could be potential molecular targets for ischemic diseases^17^. It has been suggested that Bach1 is highly expressed in ischemic areas, reducing VEGF expression by inhibiting the Wnt/β-catenin signaling pathway^18–20^. For the first time, this study found that rESWT inhibited Bach1 expression, subsequently up-regulated the Wnt/β-catenin signaling pathway, and ultimately increased VEGF expression, thereby facilitating angiogenesis.

## Materials and methods

### Cell culture

The human umbilical vein endothelial cells (HUVECs) was procured from the Cell Bank of China Medical University. HUVECs were cultivated in Dulbecco's Modified Eagle’s Medium (DMEM, Invitrogen, USA), devoid of acetone, and supplemented with 10% fetal bovine serum (FBS, Beyotime Biotechnology, Jiangsu, China). They were then incubated at 37 °C with 5% CO_2_. Cells were passaged upon reaching 90–100% confluence, typically within a period of 2 ~ 3 days. The complete medium was removed via three successive washes with PBS. One milliliter of Hyclone 0.05% trypsin containing EDTA was added to the culture flask and the cells were subsequently digested in a 37 °C cell culture incubator (Panasonic, Japan) for one minute. The cell suspension was prepared by adding one milliliter of cell culture medium, followed by centrifugation at 1000 rpm for five minutes. The supernatant was discarded, and the cells were resuspended in the medium and cultured in separate flasks.

### Oxygen glucose deprivation (OGD)/re-oxygenation

The procedure was carried out in accordance with the protocol to simulate the in vitro ischemic environment^21^. Briefly, cells that were seeded in the culture dishes were incubated at 37 °C with glucose-free DMEM medium. After 6 h, the cells were transferred back to the normal incubator and incubated with the original culture medium for re-oxygenation.

### Cells treated with rESWT

**Figure 1:**
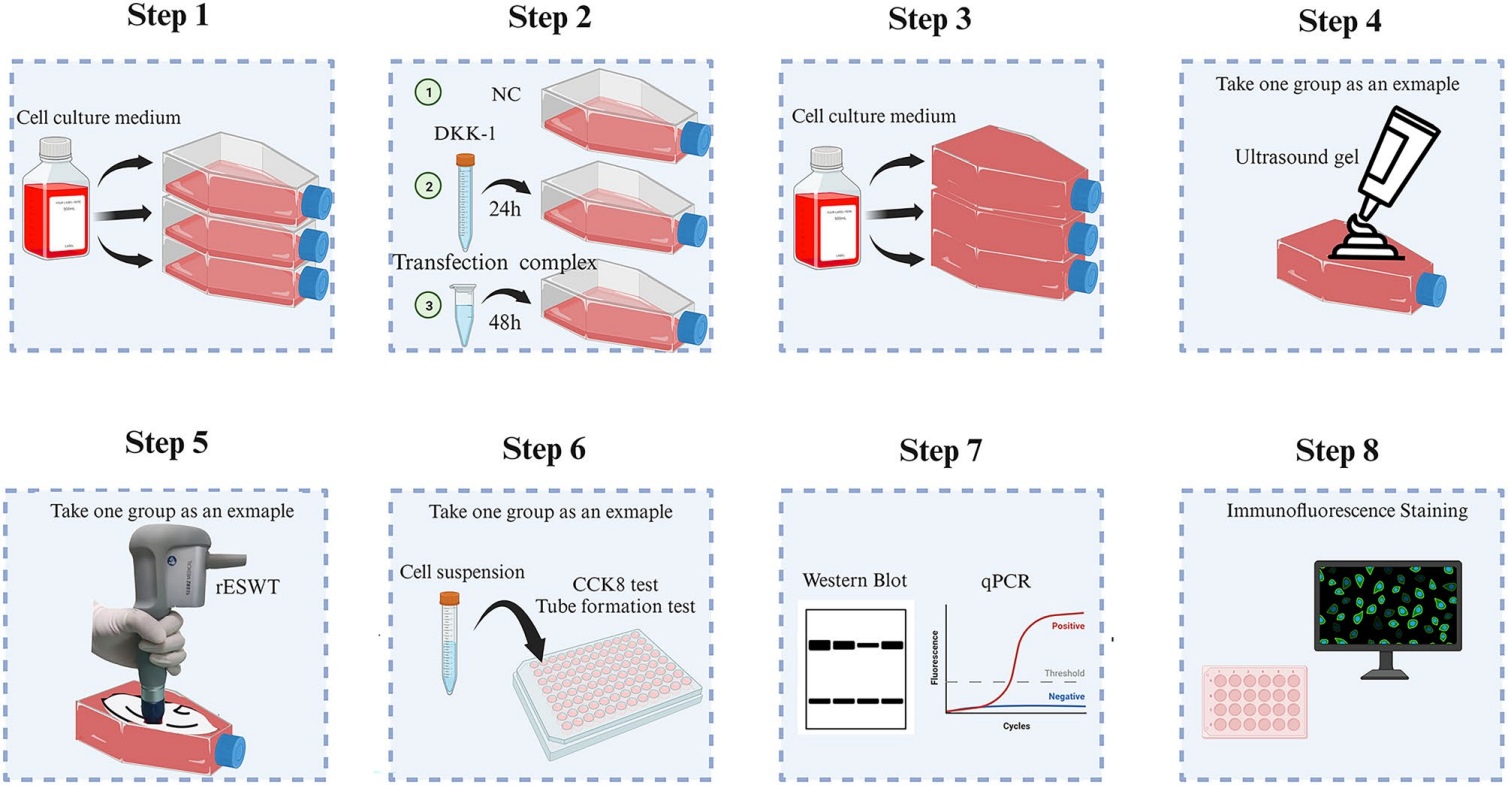
The flowchart of this research.

rESWT was applied to HUVECs that were in the exponential growth phase with a confluence of 70–80%. The culture bottle was filled with the culture medium to expel any air bubbles. The surface of the culture bottle was uniformly coated with ultrasonic gel to minimize the loss of shock wave energy at the interface between the transmitter and culture bottle. In this experiment, we used a Swiss extracorporeal shock wave device (STORZ MEDICALAG, Switzerland) with the surface probe at a dose of 2.0 Bar, 2 Hz, 200 impulses, to make vertical contact with the gel-coated surface of the cell culture bottle (Fig. 1). When using the Wnt/β-catenin signaling inhibitor Dickkopf-related protein 1 (DKK-1, MedChemExpress, NJ, USA), the plated cells were treated with 20 ng/mL DKK-1, incubated at 37 °C for 24 h, and then subjected to rESWT intervention. The modalities of rESWT application to cells are detailed in the reference^22^.

### Cell proliferation test

To investigate the influence of rESWT on HUVECs proliferation, varying doses of rESWT (surface probe, ranging from 1.0 Bar to 2.5 Bar, 200 impulses, and 2 Hz) were utilized in the cultures. The HUVECs proliferation was evaluated using the Cell Counting Kit-8 (CCK-8, Sevenbio, Beijing, China) proliferation assay. Following treatment with rESWT, cells were seeded into 96-well plates and cultivated for 24 and 36 h. Subsequently, 10 mL of CCK-8 was administered to each well, and culture plates were incubated at 37 °C for one hour. Absorbance was determined by photometry at 450 nm. A therapeutic dose of rESWT was selected as 2.0 Bar, 200 impulses, and 2 Hz. The cell proliferation assay was detailed in the referenced literature^23^.

### Cell transfection

The Bach1 overexpression plasmid and the negative control (NC) plasmid were procured from Norbeich (Hefei, China). Transient transfection of mimics and plasmid vector was conducted utilizing Lipofectamine 2000 (Thermo Fisher, CA, USA) following the manufacturer's instructions. Cells were harvested for subsequent analysis and detection 48 h post-transfection. The method of cell transfection was detailed in the referenced literature^23^.

### Tube formation test

Pipette tips, pre-chilled, were used to draw 50 μL of Matrigel assay (BD Biosciences, Bedford, MA, USA) uniformly across the base of a 96-well plate. Following distribution, 96-well plates were situated in a 37 °C cell culture incubator for 30 ~ 60 min for solidification. Test cells were digested, and cell suspensions were prepared. The cells were then counted and adjusted to 25,000 cells and 200 μL of cell suspension per well was injected into a 96-well plate containing three matrix wells. After 6 h, cells were observed using an inverted light microscope and imaging system. The total branch length of endothelial cells in each group was quantified using vascular analysis software (Angiogenesis analyze). The tube formation assay was repeated thrice. The assay was conducted as previously described^24^.

### Animals

All procedures were sanctioned by the Institutional Animal Care and Use Committee of China Medical University, adhering to the guidelines stipulated by the National Institutes of Health for the care and use of laboratory animals. The rats were maintained in a controlled environment at 23 ± 1 °C, subject to a 12-h photoperiod, with ad libitum access to food and water.

### Rat model of middle cerebral artery occlusion (MCAO)

Rats were anesthetized using a 2–3% isoflurane solution (RWD Life Science, Shenzhen, China). As previously described^25^, transient MCAO was induced. Briefly, an incision was made in the midline of the rat's neck and the skin was bluntly dissected to expose and isolate the bilateral common carotid artery (CCA). A ventral midline incision was made to expose the right common carotid artery (CCA), external carotid artery (ECA), and internal carotid artery (ICA). An intraluminal filament (Beijing Xinong Technology Co., Ltd) was inserted into the CCA lumen and carefully advanced to the ICA until it occluded the origin of the MCA. Two hours post-occlusion, reperfusion was achieved by withdrawing the filament until its tip was clear of the CCA lumen. In the Sham group, the CCA and ICA were isolated without insertion of the occluding filament.

## Experimental groups and treatment protocol

Male Sprague–Dawley rats, each weighing between 250–300 g, were randomly assigned to one of three groups: Sham (n = 3), MCAO (n = 3), or rESWT (n = 3). All experimental procedures were conducted blind to group allocation.

In the MCAO group, rats were subjected to the model procedure and then included in the study 14 days post-MCAO. For the rESWT group, radial extracorporeal shock wave therapy (rESWT) by STORZ MEDICAL AG (Switzerland) was administered to the rats post-MCAO. As reported in a prior study^12^, rESWT treatment parameters were set to a surface probe at 1.0 Bar pressure, delivering 200 impulses at a frequency of 10 Hz, initiated 72 h following MCAO. Treatments were repeated every three days for a total of 14 days. The rESWT probe was precisely oriented to target the right cerebral hemisphere of the rat, positioned perpendicularly to the cranium surface. A coupling gel was applied to both the probe and the treatment area to facilitate the rESWT procedure.

### Western blot

Cells were lysed using ice-cold radio immunoprecipitation assay (RIPA) buffer (Sevenbio, Beijing, China) supplemented with protease inhibitors (10 mg/mL aprotinin, 10 mg/mL phenylmethylsulfonyl fluoride [PMSF], and 50 mM sodium orthovanadate) to extract total protein. The protein concentration in the supernatant was determined using a BCA protein assay kit (Beyotime Biotechnology, Jiangsu, China). Protein samples (30 mg) were electrophoretically separated using sodium dodecyl sulfate–polyacrylamide gel (SDS-PAGE) and electrotransferred to a polyvinylidene fluoride (PVDF) membrane (Millipore, Shanghai, China). Non-specific binding was blocked by incubating the membranes with 5% fat-free milk in Tris-buffered saline containing 0.1% Tween-20 (TBST) at room temperature for 2 h. The membranes were then incubated overnight at 4 °C with primary antibodies as follows: Bach1 (1:1000; Abcam, USA), Wnt3a (1:1000; Abcam, USA); β-catenin (1:1000; Abcam, USA); VEGFA (1:1000; Abcam, USA); VEGFR-2 (1:1000; Abcam, USA); β-actin (1:5000; Sevenbio, Beijing, China).

The membranes were washed and subsequently incubated with HRP-conjugated secondary antibodies (Sevenbio, Beijing, China), which were diluted at a ratio of 1:3000 and maintained at room temperature for 2 h. Immunoblots were visualized using an enhanced chemiluminescence kit (ECL; Sevenbio, Beijing, China), detected by Bio-Rad Chemi-Doc (Bio-Rad, CA, USA), and subsequently normalized to β-actin levels. Prior to hybridisation with antibodies in this experiment, some blots were cut, precluding the provision of original images of the full-length blots.

### Cell immunofluorescence staining

1. A 1ml of cell suspension (containing between 10,000 and 12,000 cells) was cultured in 24-well plates with cell crawl slices. The cells reached a confluence of about 60–70% under a forward fluorescence microscope, the supernatant was then discarded, and the cells were washed three times with PBS for 3 min each time.
2. Fixation: the cells were treated with paraformaldehyde fixative for 10 to 15 min and were then washed three times.
3. Permeabilization: the cells were treated with 0.5%Trition X-100 (Sevenbio, Beijing, China) for 20 min at room temperature, and then washed three times.
4. DNA denaturation: the cells were treated with 1.5 M hydrochloric acid (diluted in PBS buffer) for 25 min at room temperature and then washed three times with PBS.
5. Blocking: the cells were treated with 10% Bovine serum albumin (BSA, Sevenbio, Beijing, China) at 37 °C for 30 min.
6. Incubation with primary antibody: the rabbit-derived Bach1 primary antibody (1:200; Abcam, USA) was mixed in a 1:1 ratio with Brdu (1:200; Abcam, USA), and the cells were incubated with the primary antibody mixture at 4℃ overnight.
7. The next day, the primary antibody was recovered and the cells were washed in PBST three times for 5 min each. The cells were then incubated with fluorescent secondary antibodies for 1 h at room temperature.
8. Nuclear staining: the cells were treated with DAPI (Sevenbio, Beijing, China) and protected from light for 5 min. The cells were then washed four times with PBST for 5 min each time.
9. Special anti-fluorescence quenching sealing tablets were added to the wells, which were then sealed.
10. The cells were observed under a fluorescence microscope (Nikon, Japan), and the images were recorded.

### Microvascular density (MVD) assay

Immunofluorescence staining was performed on fixed frozen sections to visualize blood vessels in the penumbral region, following protocols established in previous research^26^. Frozen brain tissues were sectioned at 10 μm thickness, briefly washed with PBS three times for 5 min each, and permeabilized with 0.2% Triton X-100 for 15 min at room temperature. Following further rinsing with PBS three times for 5 min each, the slides were incubated in 10% BSA for 2 h at room temperature to block nonspecific binding. The brain sections were incubated with mouse anti-CD31/PECAM-1 antibody (1:100 dilution; Novus Biologicals, USA) at 4 °C overnight and subsequently with the corresponding secondary antibodies for 1 h at room temperature. Fluorescence microscopy was employed to capture and analyze the stained sections. Slides were scanned to identify the field with the maximum microvessel density in the peri-infarct region. Images were captured, and ImageJ software was used to quantitatively measure the number of CD31-stained MVD.

### Quantitative real-time PCR

TRIzol (TransGen Biotech, Beijing, China) was utilized to isolate total RNA from HUVECs, following the manufacturer's guidelines. Subsequently, reverse transcription of 1 mg of total RNA was accomplished using a One-Step gDNA Removal and cDNA Synthesis SuperMix (TransGen Biotech, Beijing, China), in accordance with the manufacturer's instructions. The expression levels of Wnt3a and VEGF were quantified using GreenqPCR SuperMix (TransGen Biotech, Beijing, China). β-actin (Sangong Biotech, Shanghai, China) served as the endogenous control. Subsequently, Wnt3a and VEGF gene primers (Sangong Biotech, Shanghai, China) were utilized for quantitative RT-PCR. The Step ONE Plus RT-PCR System (Applied Biosystems, USA) was employed for all qRT-PCR reactions. Gene expression values were calculated using the relative quantification 2^-△△Ct^ method. The primers for Wnt3a-, VEGFA-, Bach1-, and β-actin-specific PCR were designed as follows:

Wnt3a-F: 5-TCCCACGTACTCCAACTTCCA-3,

Wnt3a-R: 5-AGCACCAGAAACACGTGCACT-3;

VEGFA-F: 5-ATCGAGTACATCTTCAAGCCAT-3,

VEGFA-R: 5-GTGAGGTTTGATCCGCATAATC-3;

Bach1-F: 5- TCTGAGTGAGAACTCGGTTTTTG-3,

Bach1-R: 5-CGCTGGTCATTAAGGCTGAGTAA-3;

β-actin-F: 5-CACCATTGGCAATGAGCGGTTC-3,

β-actin-R: 5- AGGTCTTTGCGGATGTCCACGT-3;

### Dataset collection

The expression profile dataset, GSE9877, was retrieved from the NCBI GEO database (https://www.ncbi.nlm.nih.gov/geo/query/acc.cgi?acc=GSE9877). The mRNA dataset was generated using Affymetrix U133A chips, encompassing 11 ischemic stroke (IS) samples and 27 healthy control samples.

### Differential expression analysis

Based on the platform annotation information of GSE9877, probes were converted into their corresponding gene symbols. When multiple probes corresponded to the same gene, their values were averaged to derive the gene expression value. Subsequently, the data underwent log2 transformation and normalization. The identification of differentially expressed genes (DEGs) between IS and healthy control samples was carried out using the limma package in R (www.bioconductor.org/packages/release/bioc/html/limma.html)^27^. Genes displaying a differential expression were identified based on the criterion of |log_2_(FC)|> 1 and a *P* < 0.05. Following the extraction of gene expression values in each sample, bidirectional hierarchical clustering utilizing the Euclidean distance metric was performed using the pheatmap package in R (https://cran.r-project.org/web/packages/pheatmap/index.html). Volcano plots and heat maps were generated using the ggplot2^28^ and pheatmap packages in R, respectively, to demonstrate the expression patterns of DEGs in the IS group compared to the healthy control group.

## Statistical analysis

Standard deviations for CCK-8, angiogenesis assays, Western blot, immunofluorescence staining, and qRT-PCR data were calculated using GraphPad Prism 9. An unpaired two-tailed t-test was employed for comparisons between two groups. One-way ANOVA was applied for the multiple comparison test when appropriate. Violin plots generated by the R package ggplot2 illustrated the expression pattern of Bach1 in the IS group compared with the healthy control group, based on the GSE9877 dataset. A *P*-value of less than 0.05 was considered to indicate statistical significance. All experiments were repeated at least three times to ensure biological replicability. The presented data represent the mean ± SEM derived from at least three independent experiments.

## Results

### Promotion of HUVECs Proliferation and Angiogenesis in vitro by rESWT

**Figure 2:**
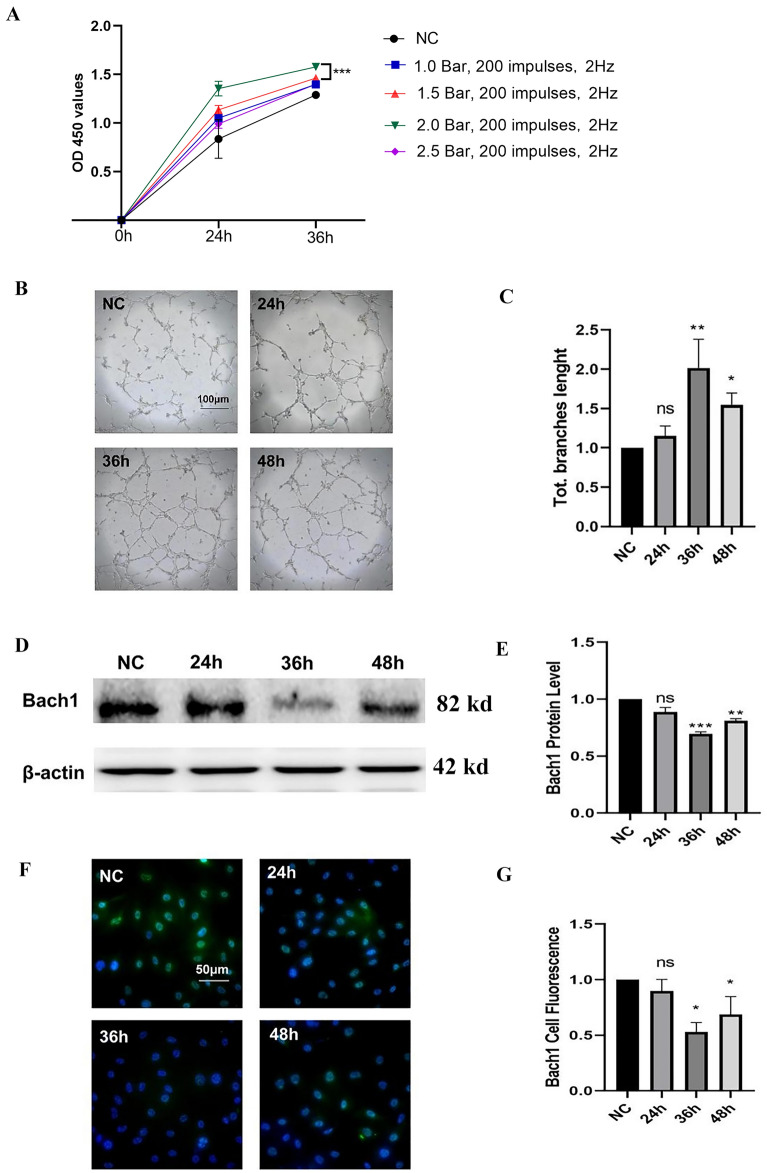
Proliferation, angiogenic tube formation, and Bach1 expression in HUVECs following rESWT. (**A**): The CCK-8 assay revealed dose-dependent proliferation of HUVECs. ***, *P* < 0.001 2.0 Bar, 200 impulses, 2 Hz vs. 1.5 Bar, 200 impulses, 2 Hz (n = 4 per group). (**B**) and (**C**): Analysis of in vitro angiogenic tube formation over time after rESWT treatment (n = 3 per group). Angiogenesis in the NC, 24 h, 36 h, 48 h groups (B; scale bar: 100 μm); Total branches length of HUVECs in NC, 24 h, 36 h and 48 h groups (**C**). **, *P* < 0.01 36h vs. NC; *, *P* < 0.05 48h vs. NC; ns denotes non-significance. (**D**) and (**E**): Western blot analysis was employed to evaluate Bach1 protein levels in the NC group without rESWT treatment, as well as in the experimental groups subjected to rESWT at various time points (24 h, 36 h, and 48 h) (n = 3 per group). ***, *P* < 0.001 36h vs. NC; **, *P* < 0.01 48h vs. NC; ns denotes non-significance. (**F**) and (**G**): Immunofluorescence staining for Bach1 in NC, 24 h, 36 h and 48 h groups (DAPI: blue fluorescence; Bach1: green fluorescence, localized to the nucleus and cytoplasm; scale bar: 50 μm) (n = 3 per group). *, *P* < 0.05 36h or 48h vs. NC, and ns denotes non-significance. All data were presented as the mean ± SEM.

In this study, we employed the CCK-8 test to evaluate the capacity of rESWT to promote HUVECs proliferation and to ascertain the most effective dose. The use of the standard probe (R15) resulted in the detachment of adherent cells; consequently, we selected the surface probe (F15) to investigate the ideal rESWT dosage for cell proliferation. The optical density (OD) values were compared between the NC group, which did not receive rESWT, and various rESWT-treated groups over a period of 0 to 36 h. rESWT was administered at intensities of 1.0, 1.5, 2.0, and 2.5 Bar, delivering 200 impulses at a frequency of 2 Hz to treat the HUVECs. As depicted in Fig. 2A, the group at 2.0 Bar exhibited the highest OD values approximately 36 h following rESWT. Consequently, subsequent experiments were conducted on HUVECs treated with 2. Bar, 200 impulses, and a frequency of 2 Hz based on these findings. To ascertain the most favorable timing of rESWT, we compared the number and the total branches length of branches in HUVECs treated for 24, 36, and 48 h with those of the NC group to assess rESWT's role in angiogenesis and identify the peak period for tube formation. Figure 2B and C demonstrated that rESWT facilitated angiogenesis in HUVECs in vitro by significantly increasing the total branches length at 36 h (*P* < 0.01) and 48 h (*P* < 0.05) as compared to the NC group. Additionally, optimal tube formation was observed at 36 h post-rESWT, as indicated by Fig. 2B and C. It should be noted that no statistical significance was observed between the 24 h treatment group and the NC group (*P* > 0.05).

### rESWT-mediated suppression of Bach1 expression

Subsequent investigations were conducted to assess the influence of rESWT on Bach1 protein expression in HUVECs. Western blot analysis was performed at 24, 36, and 48 h post-rESWT (Fig. 2D and E). Results indicated a significant reduction in Bach1 expression at 36 h (*P* < 0.001) and 48 h (*P* < 0.01) in comparison to the NC group. Similarly, Bach1 protein expression in the 36 h group exhibited the greatest decrease compared with other groups, and there was no statistically significant difference in Bach1 protein expression levels between the 24 h and NC group. Moreover, we employed immunofluorescence to assess Bach1 expression levels (Fig. 2F and G), corroborating the trend observed in Western blot analysis. Specifically, a decrease in Bach1 expression was observed at 36 h (*P* < 0.05) and 48 h (*P* < 0.05), while no significant difference was detected between the 24 h and NC groups (*P* > 0.05). The concordance of Bach1 expression results from Western blot and immunofluorescence suggests that rESWT diminishes Bach1 levels in treated HUVECs.

### rESWT-mediated up-regulation of the Wnt/β-Catenin pathway

**Figure 3:**
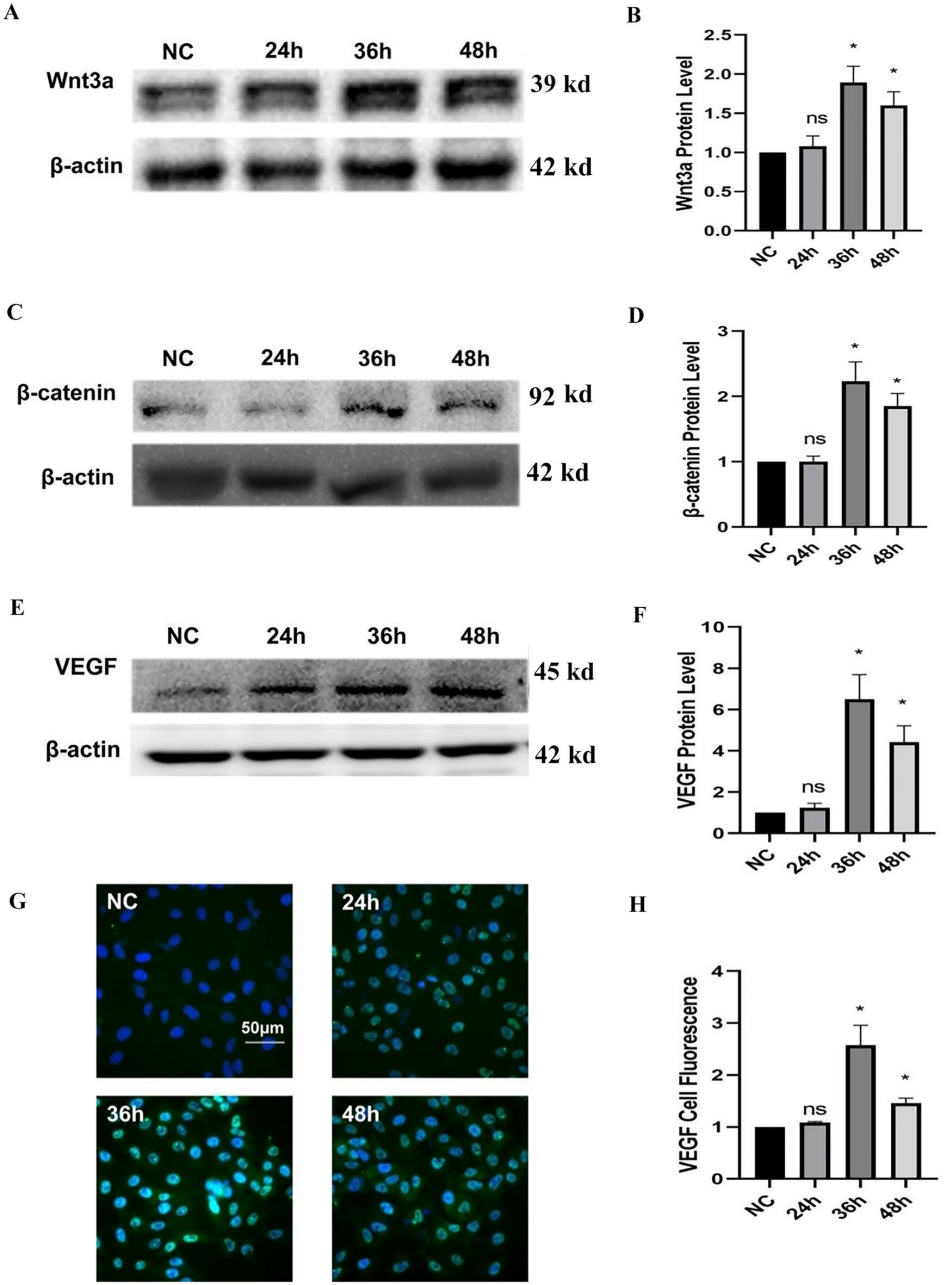
The expression of Wnt3a, β-catenin, and VEGF at different time points after rESWT. (**A**) and (**B**): Western blot analysis of Wnt3a protein expression in NC group without rESWT treatment and post-rESWT treatment at various time points (24, 36, and 48 h) (n = 3 per group). (**C**) and (**D**): Western blot assessment of VEGF protein expression in the NC group without rESWT intervention and at subsequent intervals (24, 36, and 48 h) post-rESWT (n = 3 per group). (**E**) and (**F**) Western blot assessment of VEGF protein expression in the NC group without rESWT intervention and at subsequent intervals (24, 36, and 48 h) post-rESWT (n = 3 per group). The original blots/gels were presented in Supplementary materials. (**G**) and (**H**): Immunofluorescence staining for VEGF in the NC group and groups at 24, 36, and 48 h post-rESWT (DAPI indicates nuclei with blue fluorescence; VEGF exhibits green fluorescence localized to the nucleus and cytoplasm; scale bar: 50 μm) (n = 3 per group). *, *P* < 0.05 36 h or 48 h vs. NC, and ns denotes non-significance. All data were presented as the mean ± SEM.

Subsequently, we investigated the impact of rESWT on Wnt 3a and β-catenin expression by quantifying their protein levels at 24-, 36-, and 48 h post-treatment. Figure 3A–D demonstrate an elevation in Wnt3a and β-catenin protein levels at 36 and 48 h compared to the NC group (*P* < 0.05). However, as depicted in Fig. 3, no statistically significant difference in Wnt3a and β-catenin protein expression was observed between the 24 h time point and the NC group (*P* > 0.05).

### Up-regulation of VEGF expression induced by rESWT

We also assessed VEGF protein expression levels at 24, 36, and 48 h post-rESWT and compared them with those in the NC groups. Western blot analysis, demonstrated in Fig. 3E and F, revealed that VEGF protein expression was elevated at 36 and 48 h compared to the NC group (*P* < 0.05), with peak expression observed at 36 h post-rESWT. Similarly, VEGF expression at 24 h did not differ significantly from that observed in the NC group (*P* > 0.05).

Immunofluorescence analysis also confirmed this trend of VEGF expression, as depicted in Fig. 3G and H, with significant differences between the 36 and 48 h groups and the NC group (*P* < 0.05), substantiating the up-regulation of VEGF by rESWT. Immunofluorescence findings also indicated no significant difference in VEGF expression between the 24 h group and the NC group (*P* > 0.05), as illustrated in Fig. 3G and H.

### rESWT restoring expression of Wnt3a, β-catenin, and VEGF inhibited by *Down-regulating Bach1 expression*

**Figure 4:**
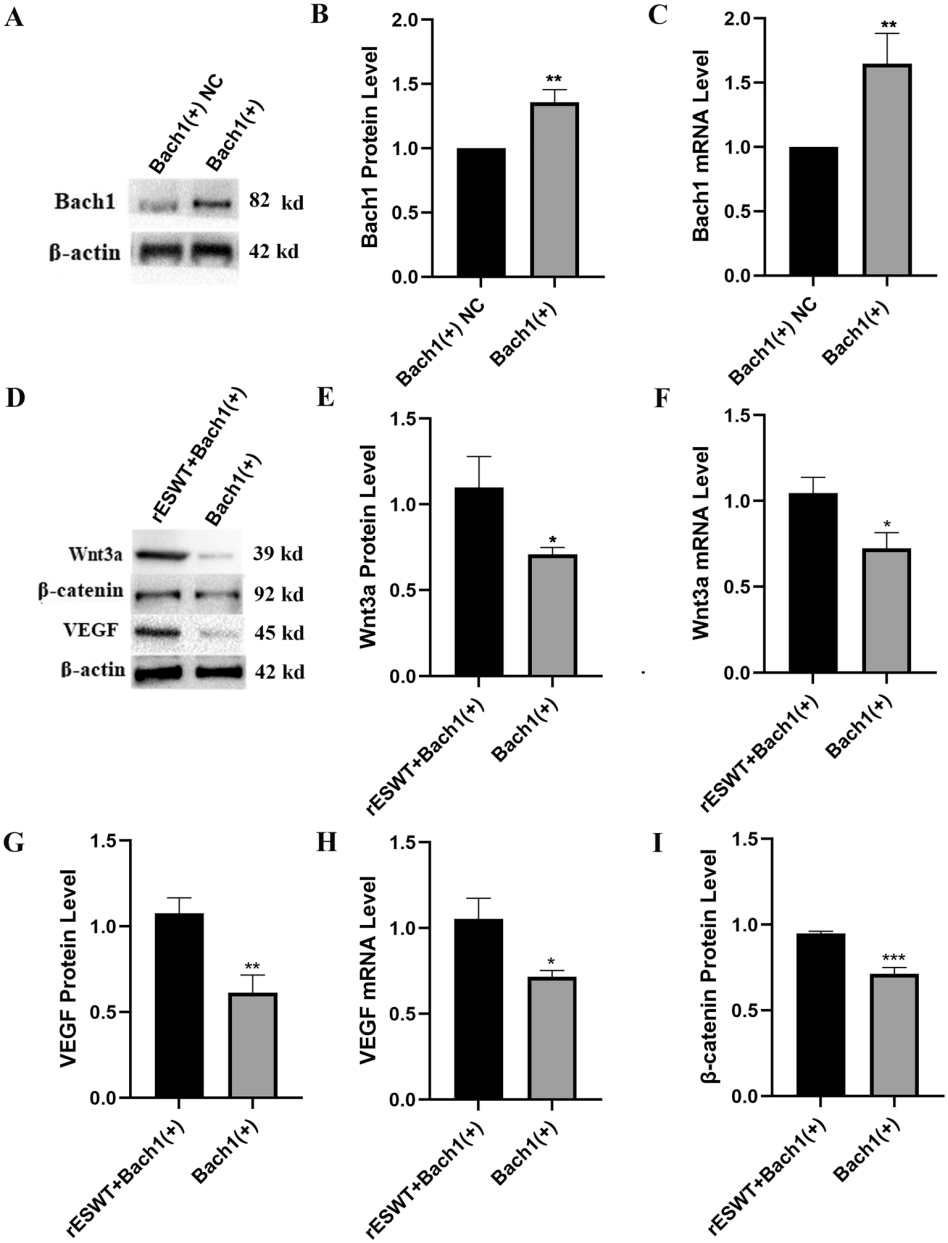
Differential expression of Bach1, Wnt3a, β-catenin, and VEGF following cell transfection in various groups. (**A**) and (**B**): The protein levels of Bach1 in Bach1 ( +) NC and Bach1 ( +) groups. **, *P* < 0.01 Bach1 ( +) vs. Bach1 ( +) NC; n = 3 per group. (**C**): Comparison of Bach1 mRNA levels between Bach1 ( +) NC and Bach1 ( +) groups. **, *P* < 0.01 Bach1 ( +) vs. Bach1 ( +) NC; n = 3 per group. (**D**): Quantitative assessment of Wnt3a, β-catenin, and VEGF protein expression in rESWT + Bach1( +) and Bach1 ( +) groups (n = 3 per group). The corresponding blots and gels can be found in the Supplementary materials. (**E**) and (**F**): Analysis of Wnt3a protein and mRNA levels in rESWT + Bach1( +) and Bach1 ( +) groups (n = 3 per group). *, *P* < 0.05 Bach1 ( +) vs. rESWT + Bach1( +). (**G**) and (**H**): VEGF protein and mRNA expression profiles in rESWT + Bach1( +) and Bach1 ( +) groups (n = 3 per group). *, *P* < 0.05 Bach1 ( +) vs. rESWT + Bach1( +); **, *P* < 0.01 Bach1 ( +) vs. rESWT + Bach1( +). (**I**): β-catenin protein levels in rESWT + Bach1( +) and Bach1 ( +) groups (n = 3 per group). ***, *P* < 0.001 Bach1 ( +) vs. rESWT + Bach1( +). All data were presented as the mean ± SEM.

Ischemia is known to elevate Bach1 expression, which consequently suppresses the Wnt/β-catenin pathway and diminishes VEGF expression. To simulate ischemia-induced Bach1 up-regulation, we introduced an overexpression plasmid for Bach1 (Bach1( +)) into HUVECs. The cells transfected with the Bach1 overexpression plasmid were termed the Bach1 ( +) cells. For comparative analysis, we transfected a negative control plasmid into HUVECs, creating the NC cell group. The expectation is that Bach1( +) cells will exhibit elevated Bach1 expression levels, owing to the introduction of a Bach1-overexpressing plasmid, relative to the NC cells. Western blot and qRT-PCR analyses (Fig. 4A,B, and C) revealed that both protein and mRNA levels of Bach1 were significantly elevated in Bach1( +) cells compared to NC cells, confirming successful transfection of the overexpression plasmid into HUVECs (*P* < 0.01).

Subsequently, we segregated the Bach1( +) cells into two groups: the rESWT-treated group (rESWT + Bach1( +)) and the untreated group (Bach1( +)-only). After 36 h, it was observed that protein levels of Wnt3a (*P* < 0.05), β-catenin (*P* < 0.001), and VEGF (*P* < 0.01), as well as mRNA levels of Wnt3a and VEGF (*P* < 0.05 for both), were significantly reduced in the Bach1( +) group compared to the rESWT + Bach1( +) group. Consequently, comparison between the rESWT + Bach1( +) and Bach1( +) groups revealed that rESWT could counteract the suppressive effect of Bach1 on Wnt3a, β-catenin, and VEGF expression. These findings indicate that rESWT mediates the expression of Wnt3a, β-catenin, and VEGF by attenuating Bach1 expression, as depicted in Fig. 4.

### VEGF expression mediated by rESWT through the Wnt/β-catenin pathway

**Figure 5:**
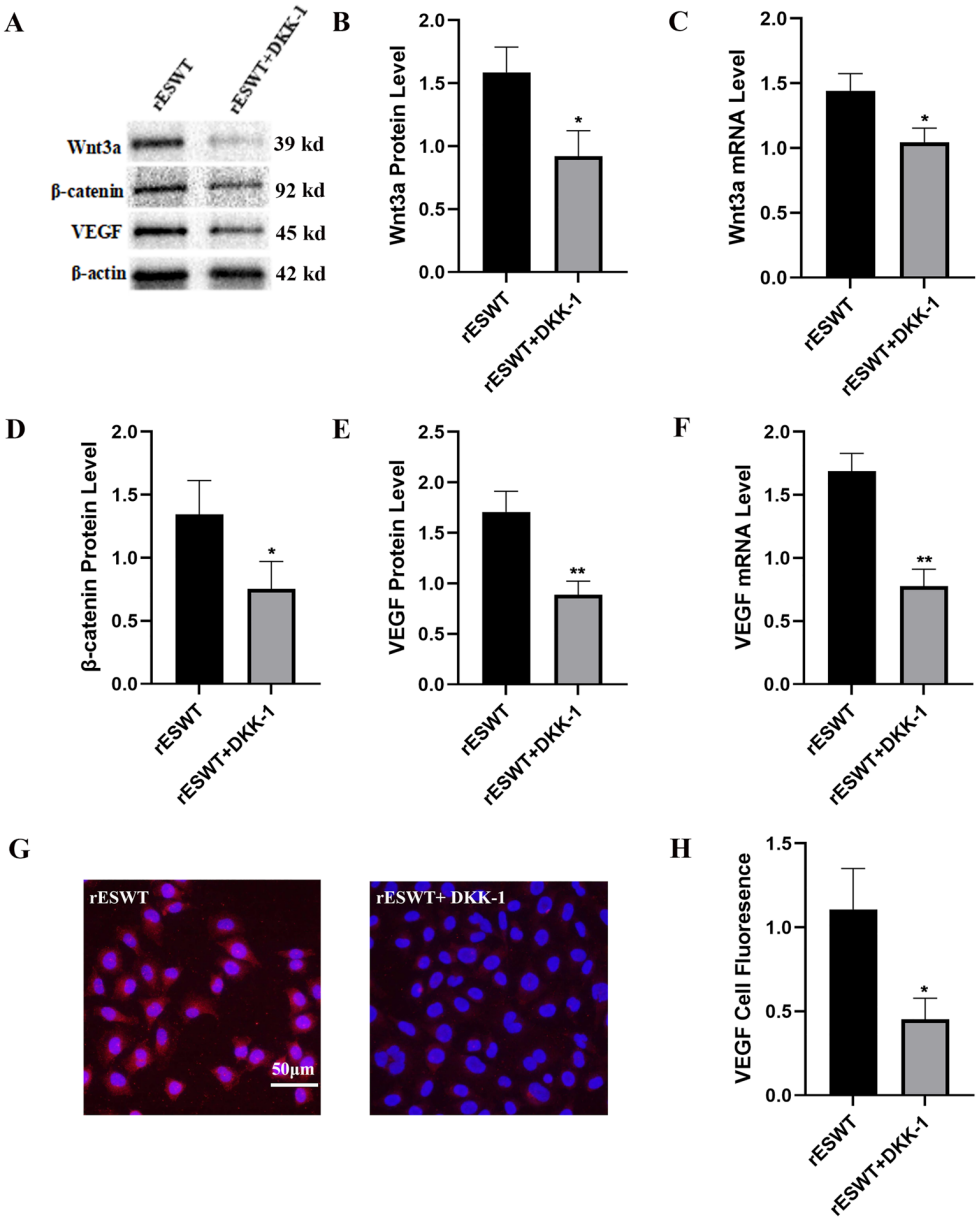
Expression profiles of Wnt3a, β-catenin, and VEGF in the rESWT and rESWT + DKK-1 groups. (**A**): Western blot analysis depicting the protein expression levels of Wnt3a, β-catenin, and VEGF in the rESWT and the rESWT + DKK-1 groups (n = 3 per group). Original blots and gels can be found in the Supplementary materials. (**B**) and (**C**): Wnt3a protein and mRNA levels in the rESWT and rESWT + DKK-1 groups (n = 3 per group). (**D**): β-catenin protein concentrations in the rESWT and rESWT + DKK-1 groups (n = 3 per group). (**E**) and (**F**): VEGF protein and mRNA expression levels in the rESWT and rESWT + DKK-1 groups (n = 3 per group). (**G**) and (**H**): VEGF immunofluorescent staining in the rESWT and rESWT + DKK-1 groups (DAPI: blue; VEGF: red, localized to the nucleus and cytoplasm; scale bar: 20 μm) (n = 3 per group). *, *P* < 0.05 rESWT + DKK-1 vs. rESWT; **, *P* < 0.01 rESWT + DKK-1 vs. rESWT. All data were presented as the mean ± SEM.

To investigate the influence of rESWT on the Wnt/β-catenin pathway and subsequent VEGF expression, cells were assigned to either the rESWT group or the rESWT group with DKK-1, a Wnt/β-catenin pathway inhibitor, to compare the expression of Wnt3a, β-catenin, and VEGF. The rESWT group exhibited significantly elevated levels of Wnt3a (*P* < 0.05), β-catenin (*P* < 0.05), and VEGF (*P* < 0.01) proteins compared to the rESWT + DKK-1 group. Likewise, mRNA levels of Wnt3a (*P* < 0.05) and VEGF (*P* < 0.01) were elevated in the rESWT group compared to the rESWT + DKK-1 group. Immunofluorescence analysis revealed that VEGF expression was more pronounced in the rESWT group than in the rESWT + DKK-1 group (*P* < 0.05), as depicted in Fig. 5.

### Elevated Bach1 gene expression in ischemic stroke patients

**Figure 6:**
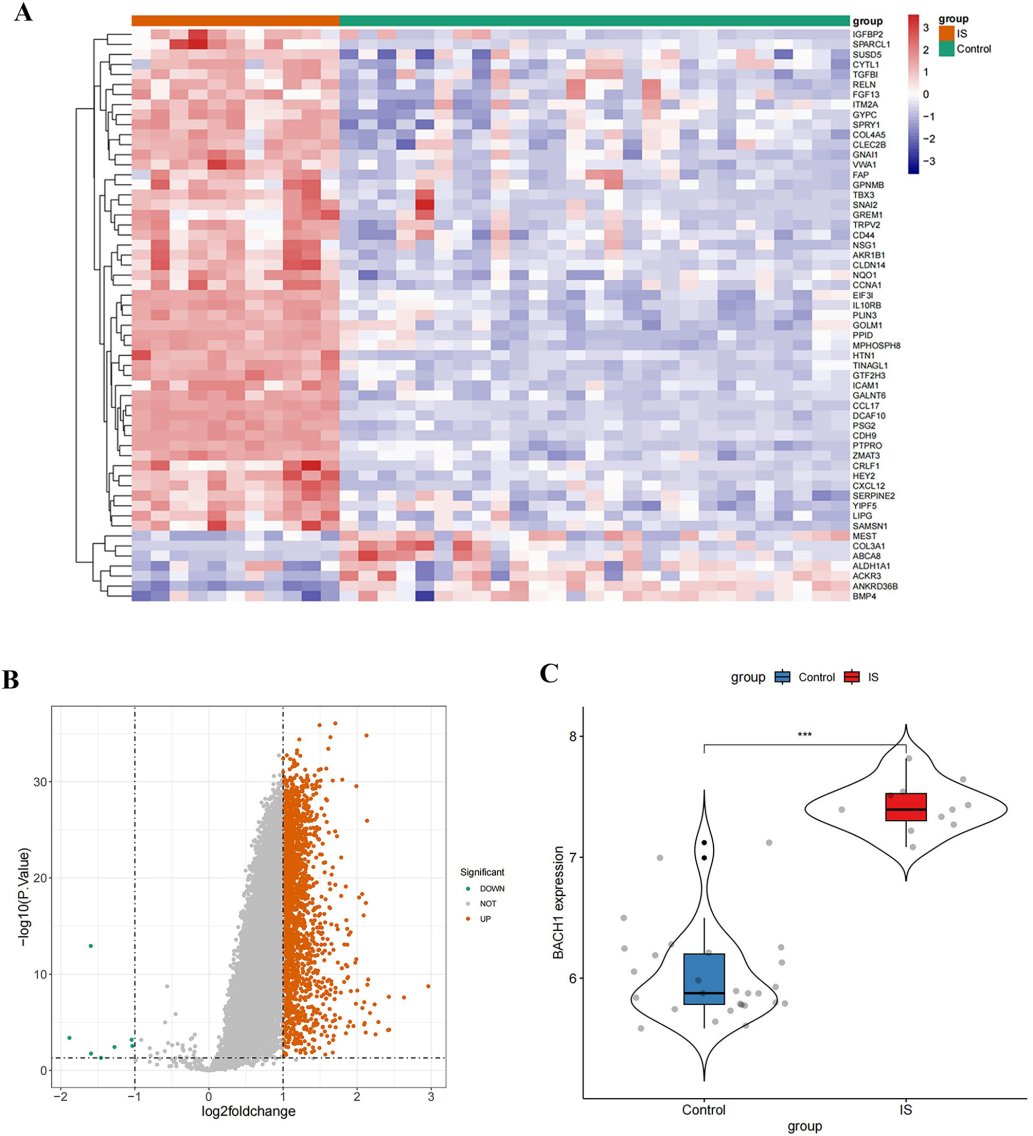
Differential expression analysis performed based on the GSE9877 dataset. (**A**): Hierarchical clustering analysis showing DEGs between IS (n = 11) and healthy control (n = 27) groups (data originally from GSE9877). (**B**): Volcano plot of GSE9877 was analyzed using |log_2_ FC|> 1 and *, *P* < 0.05. Up-regulated DEGs were shown in red and down-regulated DEGs were shown in blue. (**C**): The difference of Bach1 between 27 healthy controls and 11 IS (blue color represent healthy controls group and red colors represent IS group. ***, *P* < 0.001 IS vs. healthy control.

Differential expression analysis was conducted using gene expression profiles from the GSE9877 dataset, as shown in Fig. 6A and B. A total of 2,117 DEGs were identified, with 2110 up-regulated and 7 down-regulated. The heatmap illustrated the expression patterns of the top 50 up-regulated genes and all down-regulated genes, comparing the IS group (n = 7) to the healthy control group (n = 27). The Bach1 gene exhibited elevated expression in the IS group compared to the control group (Fig. 6C, *p* < 0.001). These findings lay the groundwork for the transfection of the Bach1 plasmid, to emulate the ischemic environment, and offer theoretical support for creating an OGD/re-oxygenation model with elevated Bach1 expression.

### rESWT suppresses Bach1 and enhances angiogenesis in ischemic environment

**Figure 7:**
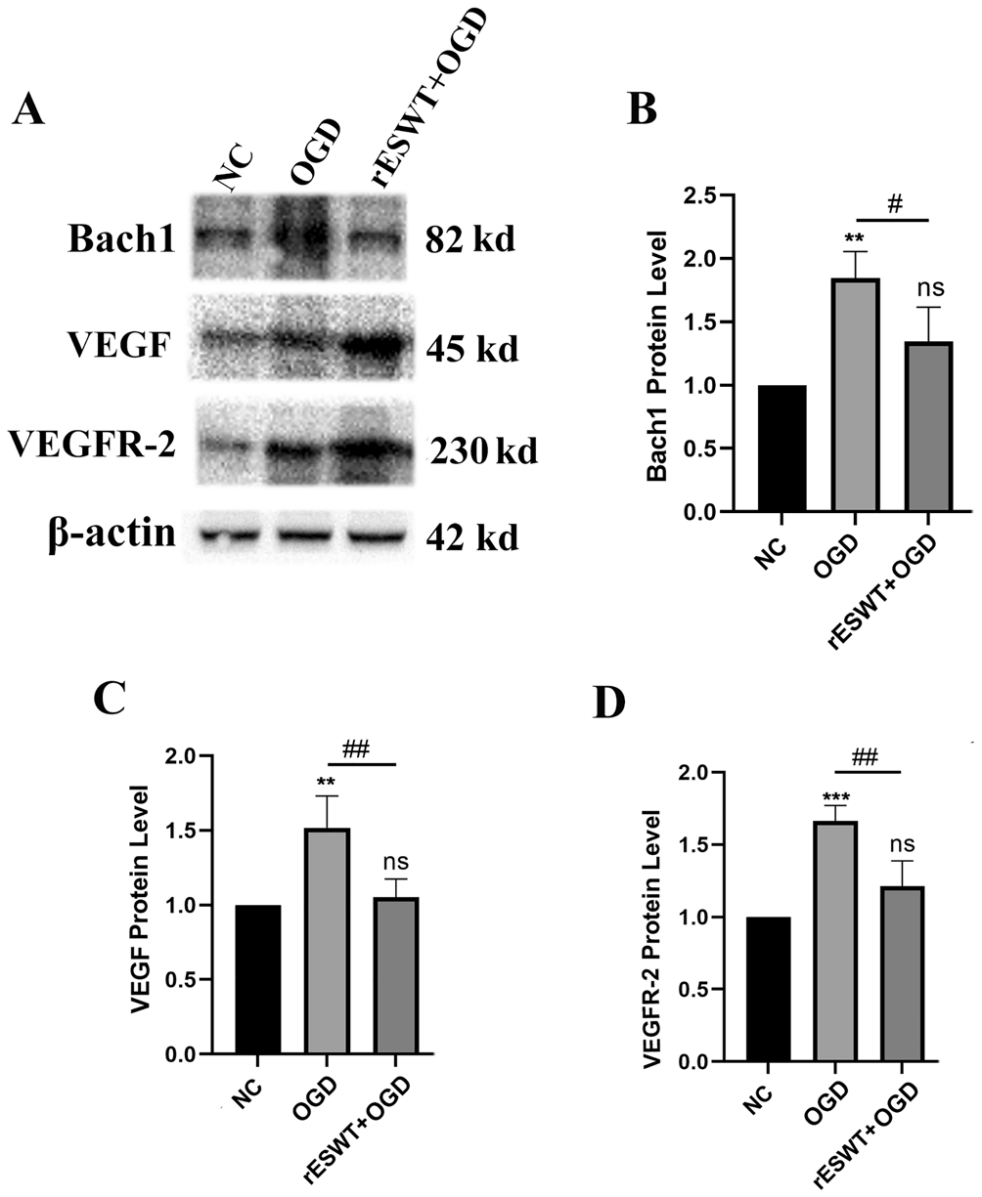
Comparative expression of Bach1, VEGF, and VEGFR-2 in the NC group, OGD group, and rESWT + OGD groups. (**A**): The protein expression levels of the Bach1, VEGF and VEGFR-2 were detected by Western blot. The original blots/gels are presented in Supplementary materials. (**B**–**D**): The protein level was quantified and analyzed (n = 3). **, *P* < 0.01 and ***, *P* < 0.001 compared with the NC group; #, *P* < 0.05 and ##, *P* < 0.01 compared with OGD group; ns denotes no statistical significance between groups. All data were presented as the mean ± SEM.

To mimic ischemic conditions in vitro, we developed an OGD model. The experiment revealed a significant up-regulation of Bach1 expression (*P* < 0.01), as well as augmented levels of VEGF (*P* < 0.01) and its receptor, VEGFR-2 (*P* < 0.001), in OGD group. Bach1 expression was significantly reduced in the rESWT + OGD group compared to the OGD group (*P* < 0.05). Following rESWT treatment, there was a significant increase in VEGF and VEGFR-2 expression compared to the OGD group (***P*** < 0.01), indicating that rESWT fosters angiogenesis in ischemic conditions, as depicted in Fig. 7.

### rESWT promotes angiogenesis in MCAO rats

**Figure 8:**
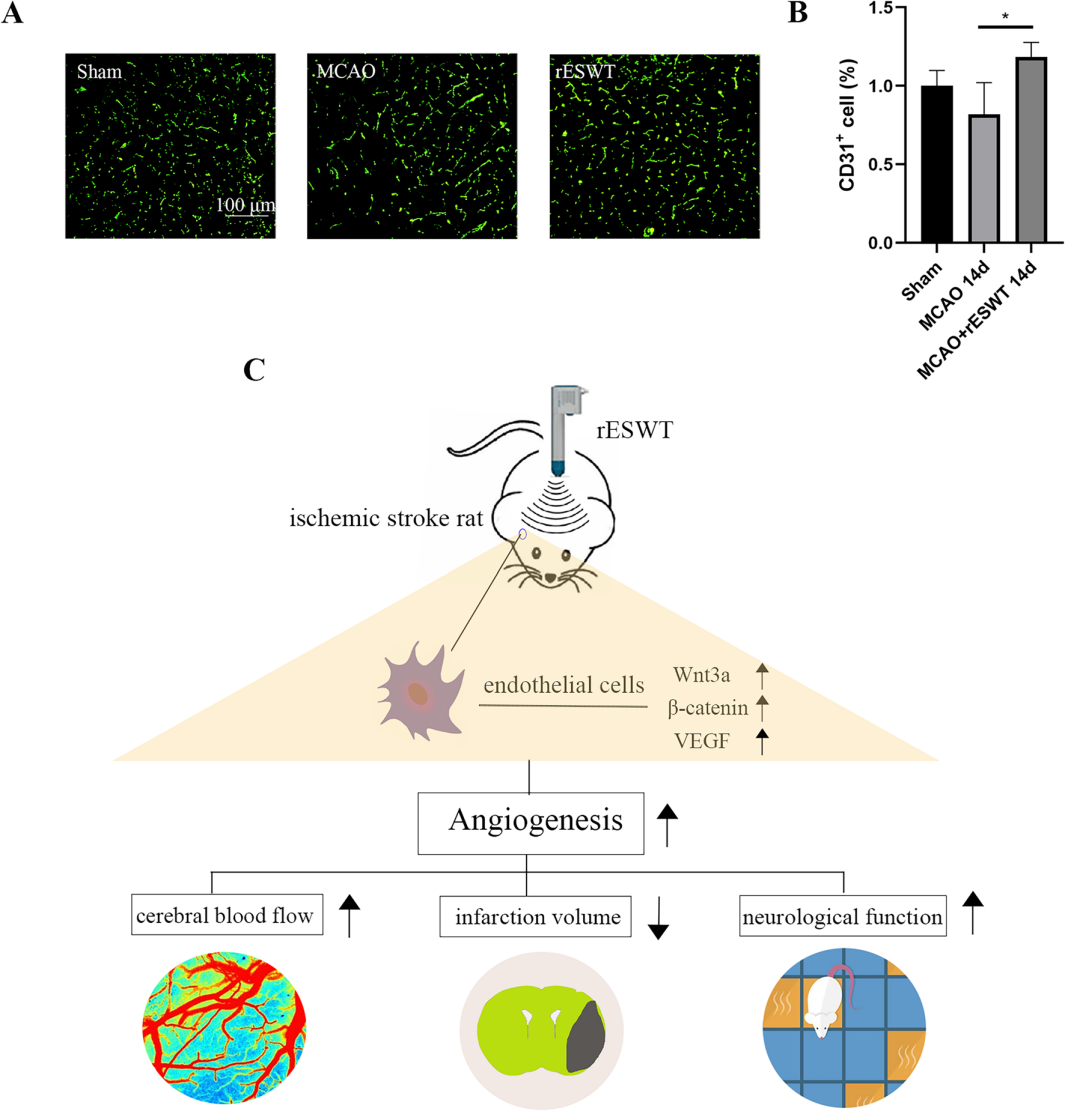
In vivo experiments demonstrated the effect and mechanism of rESWT in MCAO. (**A**) and (**B**): The number of microvessels positive for the CD31 was detected by immunofluorescence staining (n = 3). *, *P* < 0.05 compared with MCAO group. All data were presented as the mean ± SEM. (**C**): Depiction of the effects and underlying mechanisms of rESWT on rats subjected to MCAO according to our previous experiments.

The MCAO rat model was successfully established, with rESWT intervention administered 72 h post-MCAO. Immunofluorescence staining facilitated the comparison of CD31-positive microvessel counts among the Sham, MCAO, and rESWT groups. The analysis revealed an increase in CD31-positive microvessels in the rESWT group compared to the MCAO group at 14 days post-MCAO (*P* < 0.05, Fig. 8A,B).

## Discussion

rESW, also known as pneumatically-driven ballistic extracorporeal shock wave, is characterized by a stable output waveform and consistent therapeutic effects^29^. rESWT has been employed in various systemic diseases, demonstrating biological effects such as enhanced blood circulation, pain relief, and tissue repair^7^. ESWT has been shown to modulate VEGF, thereby accelerating the healing of refractory wounds, including diabetic foot ulcers, by improving blood flow and angiogenesis^30,31^. In the ischemic cardiomyopathy, ESWT intervention significantly increased endothelial progenitor cells, angiopoietin-3, and VEGF, thereby enhancing blood perfusion^32^. ESWT effectively treat both early and late-stage osteonecrosis of the femoral head by promoting angiogenesis^33,34^, which prevents the collapse of the femoral head due to increased VEGF expression and activation of the nitric oxide signaling pathway in the necrotic subchondral bone^33^. For ischemic kidney diseases, low-energy shockwave therapy up-regulated angiogenic factors, such as VEGF, endothelial nitric oxide synthase and angiopoietin-1, promoting microvascular maturation and stabilization, thereby improving kidney function^35^. Furthermore, additional research has indicated that ESWT effectively promotes angiogenesis by up-regulating VEGF expression^36,37^. Additionally, prior experiments have indicated that rESWT enhances the Wnt/β-catenin signaling pathway in MCAO rats and neural stem cells^12,22^. This study is the first to demonstrate that rESWT inhibits the expression of Bach1, thereby up-regulating the Wnt/β-catenin signaling pathway and increasing VEGF expression, which promotes angiogenesis in HUVECs in vitro.

It should be noted that HUVECs were utilized in this study due to their resemblance to cerebral endothelial cells and their capability for tubulogenesis^38^. Although research has examined the tubulogenesis of HUVECs, there are scant investigations employing rESWT to elucidate its effect and underlying mechanisms on HUVECs angiogenesis. Intervention with ESWT in HUVECs lays the groundwork for the application of rESWT in cerebral vasculature and the enhancement of cerebral blood circulation. This experiment demonstrated that rESWT administered at optimal doses significantly promoted HUVECs proliferation and tubulogenesis. Optimal proliferation was observed when rESWT was administered at 2.0 Bar, with 200 impulses and a frequency of 2 Hz. Additionally, assessments of tubulogenesis and proliferation indicated that the 36 h time point was most conducive for rESWT efficacy. It is evident that selecting the optimal dose of rESWT is of paramount importance for clinical applications. High-energy rESWT may induce damage to neuronal cells, whereas insufficient dosage may attenuate its therapeutic efficacy.

Bach 1 is a transcriptional regulator that belongs to the basic region leucine zipper (bZIP) factor family, including the cap 'n' collar subfamily^39^. Immunofluorescence assays in this study indicated that Bach1 localizes to both the cytoplasm and the nucleus, predominantly residing in the latter. In mice subjected to MCAO, neurological deficits were observed concomitant with the up-regulation of Bach1 expression^18^. Here, we employed bioinformatics techniques to demonstrate that Bach1 was overexpressed in patients suffering from ischemic stroke. Research has revealed that Bach1 suppresses heme oxygenase-1 activity, augments mitochondrial ROS generation in cells, and inhibits angiogenesis^40^. Down-regulation of Bach1 in human microvascular endothelial cells has been found to enhance angiogenesis^41^. Given rESWT's angiogenic effects, potential associations between rESWT and Bach1 were investigated by measuring Bach1 expression in HUVECs. Results indicated a tendency for Bach1 levels to decline following rESWT, which correlated with enhanced angiogenesis as evidenced by tube formation assays. Considering the inhibitory role of Bach1 on angiogenesis, rESWT is speculated to facilitate tube formation by downregulating Bach1 expression.

Bach1 modulates Wnt/β-catenin signaling, a pathway implicated in angiogenesis within brain, normal peripheral, and cancerous tissues^42^. Wnt3a can bind to and activate certain proteins, thereby inhibiting β-catenin phosphorylation. This inhibition allows β-catenin to accumulate in the cytoplasm and subsequently translocate into the nucleus, where it inversely modulates the expression of Wnt target genes^43^. During this process, β-catenin is identified as a pivotal element in controlling downstream target genes of the Wnt/β-catenin pathway^44,45^. rESWT is primarily employed as an angiogenetic therapy to modulate HUVEC activity, resulting in the up-regulation of Wnt3a and β-catenin. Moreover, introducing an overexpressed Bach1 plasmid into HUVECs, rESWT can restore the diminished expression levels of Wnt3a and β-catenin caused by Bach1 overexpression. Concurrently, an increase in VEGF levels was observed following rESWT application; however, VEGF expression decreased with Bach1 overexpression and Wnt/β-catenin pathway inhibition, a reduction that rESWT could subsequently mitigate.

The VEGF family comprises VEGFA, VEGFB, VEGFC, VEGFD, and VEGFE^46^. Typically, VEGF refers to VEGFA^42^. VEGF may confer neuroprotective effects by facilitating cerebrovascular repair following cerebral ischemia^47^. The up-regulation of VEGF expression is widely acknowledged to enhance angiogenesis across various experimental models^48^. Moreover, stroke patients exhibiting elevated vascular density in the ischemic cortex, coupled with increased VEGF expression, tend to have lower recurrence rates and higher survival rates. This indicates that enhancing cerebral angiogenesis may be advantageous in treating ischemic stroke^49^. VEGFR-2 is an important modulator of the signaling pathways involved in VEGFA mediated angiogenesis within the central nervous system (CNS)^50^. Utilizing an OGD/re-generation model to replicate the ischemic environment, our study revealed that rESWT not only attenuated the ischemia-induced up-regulation of Bach1 expression. Additionally, rESWT was found to stimulate the expression of VEGF and VEGFR-2 following ischemia, thereby facilitating angiogenesis in the post-ischemic phase.

The MCAO model was concurrently established to evaluate rESWT's promotion of angiogenesis in vivo. Our findings demonstrate a marked increase in CD31-positive microvessel density in MCAO rats after rESWT, confirming our previous in vivo observations. Previously, we developed a rat MCAO model and applied rESWT for ischemic stroke treatment (Fig. 8C). Following rESWT, there was an upsurge in the protein and gene expression levels of Wnt3a and β-catenin in the brains of rats with ischemic stroke. Western blot, qRT-PCR, and immunofluorescence analyses all revealed augmented VEGF expression in the cortical region post-stroke, attributed to rESWT^12^. Furthermore, laser speckle imaging revealed improvements in cerebral blood flow following rESWT^12^. Meanwhile, studies demonstrated that ESWT could augment CD31 and VEGF expression, thus promoting angiogenesis in the brain and ultimately diminishing the infarct volume in ischemic stroke rats^51^. The mechanisms elucidated by the animal experiments were depicted in Fig. 8C. Recent studies have also revealed that Bach1 expression is elevated in the brains of MCAO rats, and that suppressing Bach1 expression can mitigate cell damage induced by stroke in these brains^52^. Subsequent in vivo experiments are necessary to confirm the effect of rESWT on Bach1 expression in ischemic stroke rat brains and to develop a model of Bach1 overexpression, enabling a more detailed assessment of the interactions among molecular pathways. However, the application of ESWT to the human brain has been limited to isolated cases^53,54^, and to date, there are no global clinical trials investigating the effects of rESWT on humans with ischemic stroke. Thus, this research lays the groundwork for potential future applications of ESWT in treating human brain disorders.

## Conclusions

**Figure 9:**
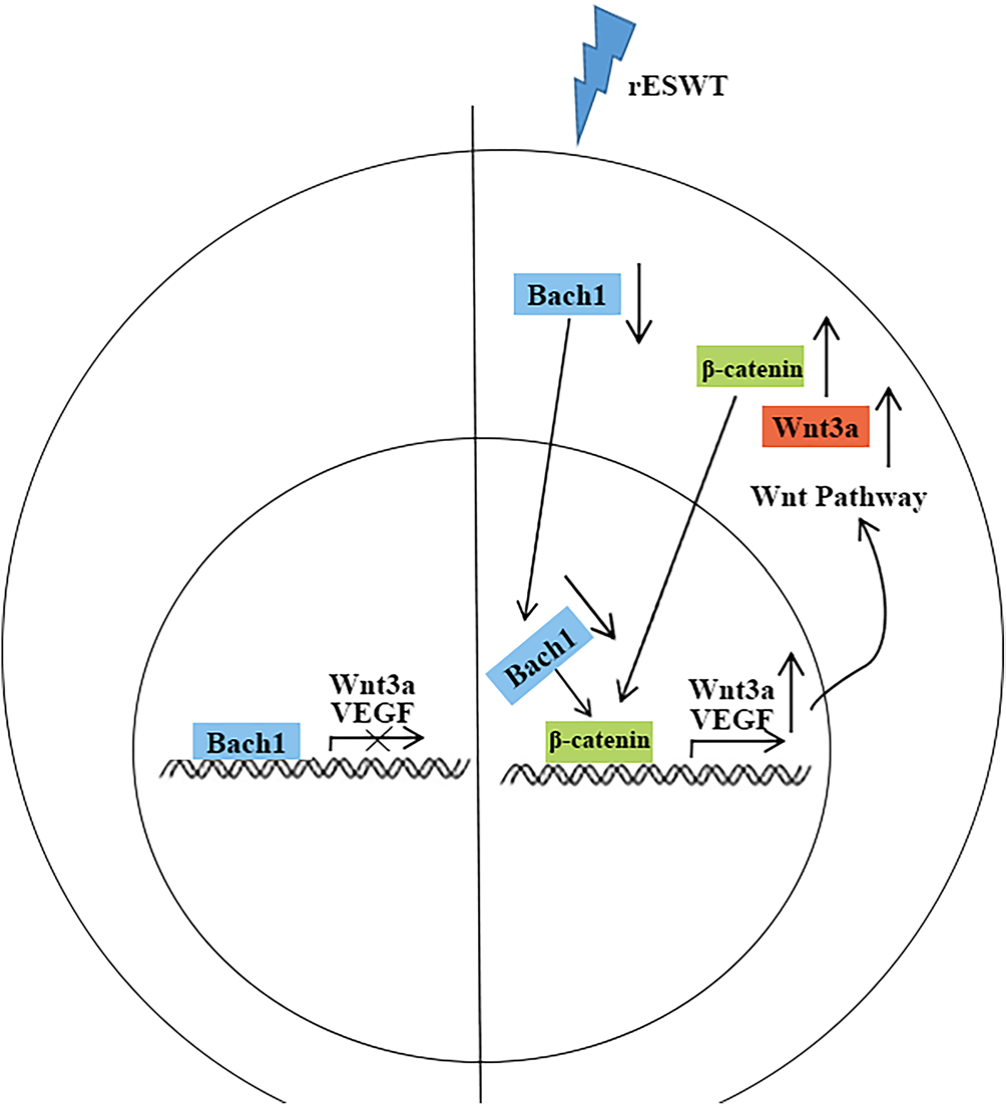
Schematic representation of the rESWT mechanism investigated in this study.

In summary, rESWT effectively suppressed Bach1 expression, which in turn reversed the down-regulation of Wnt3a and β-catenin. Consequently, this also reversed the down-regulation of VEGF expression, leading to enhanced angiogenesis (as depicted in Fig. 9). rESWT may exert a pivotal role by down-regulating Bach1 expression in the ischemic nervous system. The experiment was innovative as it integrated the effective physical treatment rESWT with signaling pathways and elucidated the angiogenic mechanism of rESWT, potentially aiding in the recovery from cerebral ischemia.

## Data Availability

The datasets produced or analyzed in this study are accessible from the corresponding author upon reasonable request. Furthermore, the GSE9877 dataset employed in this study is accessible in the GEO database, available at https://www.ncbi.nlm.nih.gov/geo/query/acc.cgi?acc=GSE9877. Additionally, all analyses conducted with R software on the GSE9877 dataset in this study can be provided as original code by the corresponding author, given a reasonable request.

## Supplementary Figures

Supplementary Figures.

## Abbreviations

HUVECs: Human umbilical vein endothelial cells
rESWT: Radial extracorporeal shock wave therapy
Bach1: BTB and CNC homology 1
BTB: Broad complex, tramtrack, bric-a-brac
CNC: Cap'n'collar
DMEM: Dulbecco's Modified Eagle’s Medium
FBS: Fetal bovine serum
OGD: Oxygenglucose deprivation
DKK-1: Dickkopf-related protein 1
CCK-8: Cell Counting Kit-8
PMSF: Phenylmethylsulfonyl fluoride
SDS-PAGE: Sodium dodecyl sulfate–polyacrylamide gel electrophoresis
PVDF: Polyvinylidene fluoride
BSA: Bovine serum albumin
IS: Ischemic stroke
MCAO: Middle cerebral artery occlusion
ECA: External carotid artery
ICA: Internal carotid artery
CCA: Common carotid artery
