# Supplementary material for: rESWT promoted angiogenesis via Bach1/Wnt/β-catenin signaling pathway
Source: Sci Rep. 2024 May 22;14:11733. doi: 10.1038/s41598-024-62582-2 (PMC11111732; doi:10.1038/s41598-024-62582-2)
Supplement: Supplementary file 1 — Supplementary Figures. [file 41598_2024_62582_MOESM1_ESM.pptx]

## Slide 1
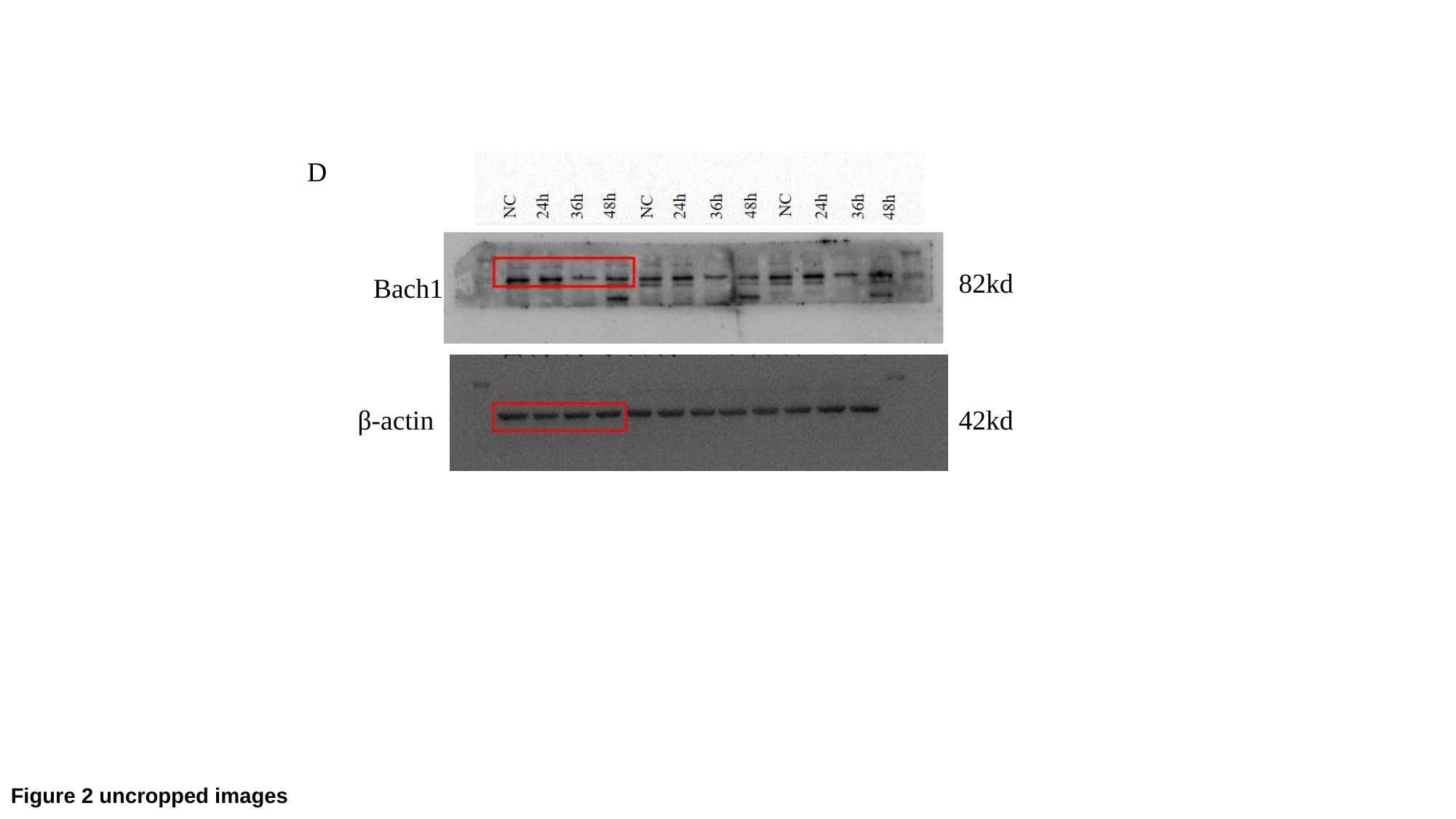

D
Bach1
82kd
β-actin
42kd
Figure 2 uncropped images

## Slide 2
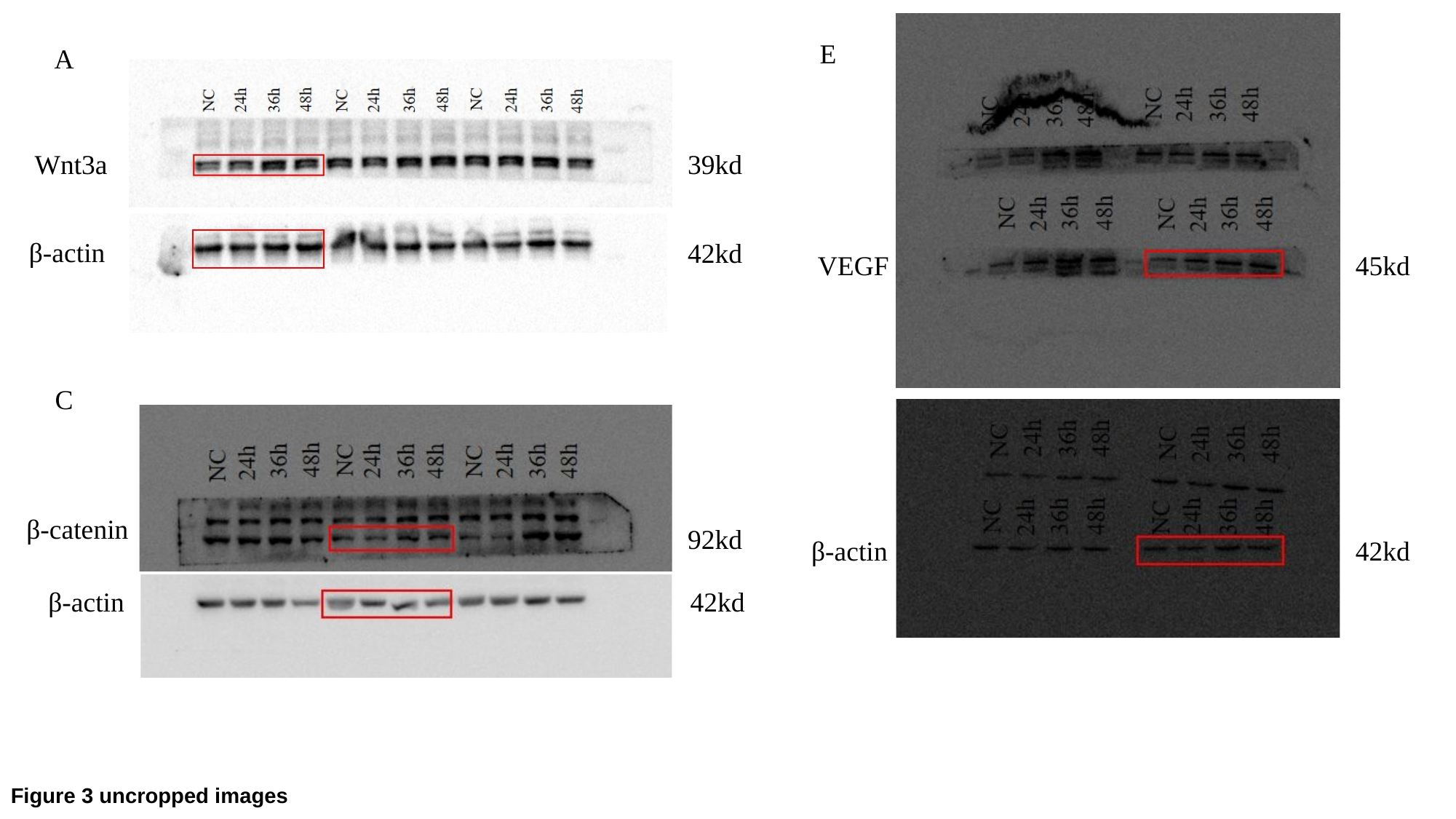

E
A
Wnt3a
39kd
β-actin
42kd
VEGF
45kd
C
β-catenin
92kd
β-actin
42kd
β-actin
42kd
Figure 3 uncropped images

## Slide 3
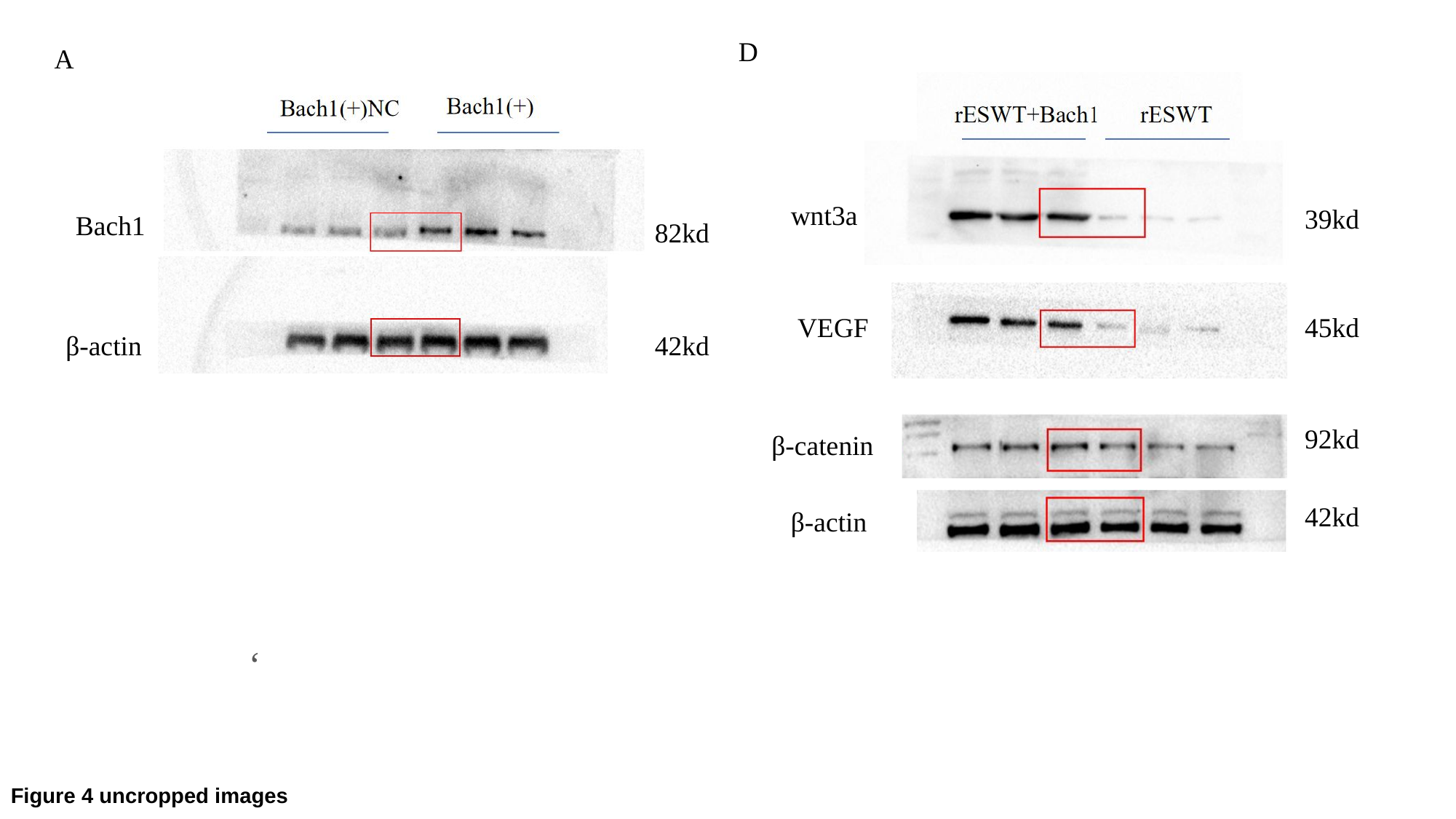

D
A
wnt3a
39kd
 Bach1
82kd
VEGF
45kd
 β-actin
42kd
92kd
β-catenin
42kd
β-actin
‘
Figure 4 uncropped images

## Slide 4
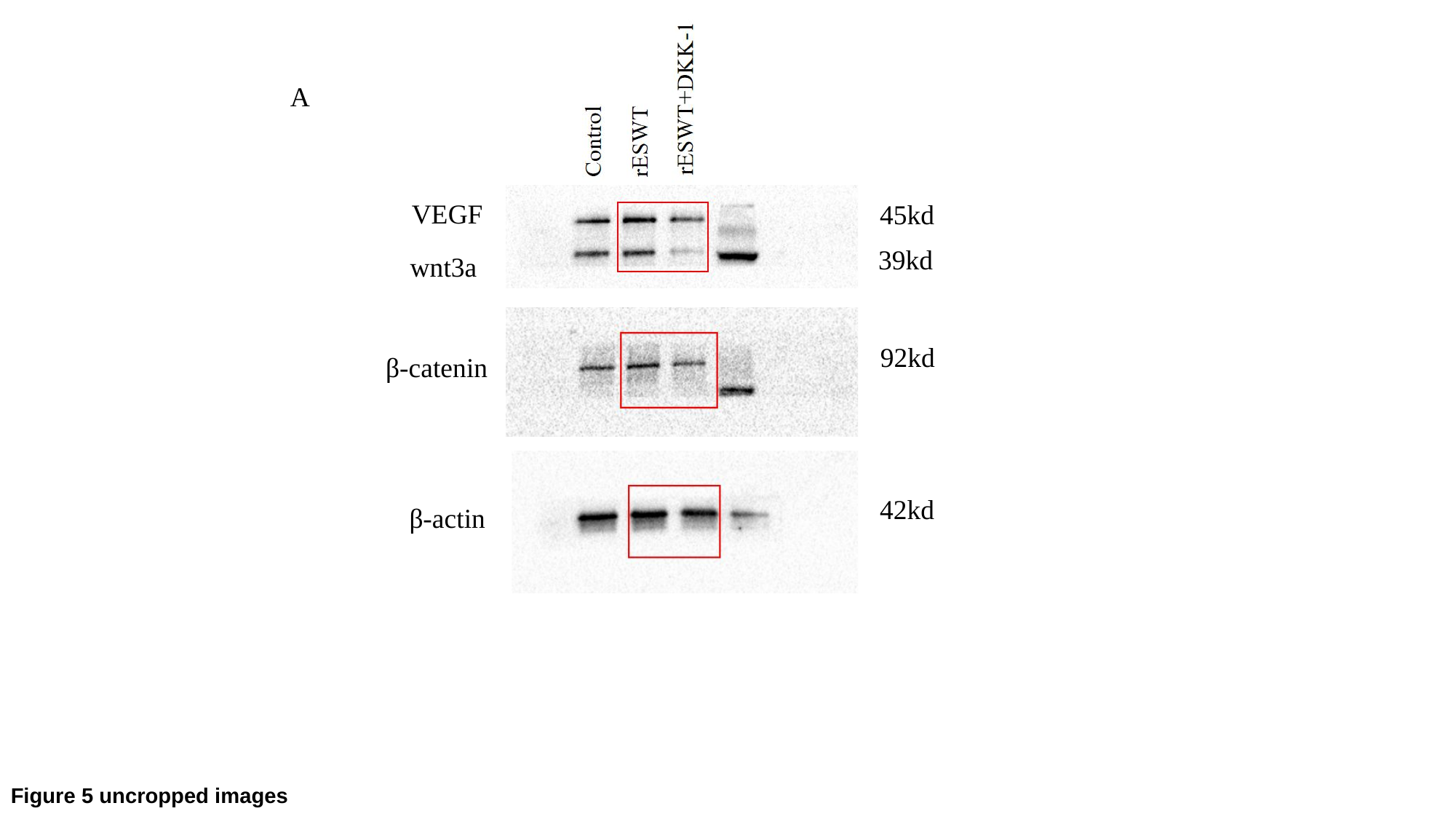

A
VEGF
45kd
39kd
wnt3a
92kd
β-catenin
42kd
β-actin
Figure 5 uncropped images

## Slide 5
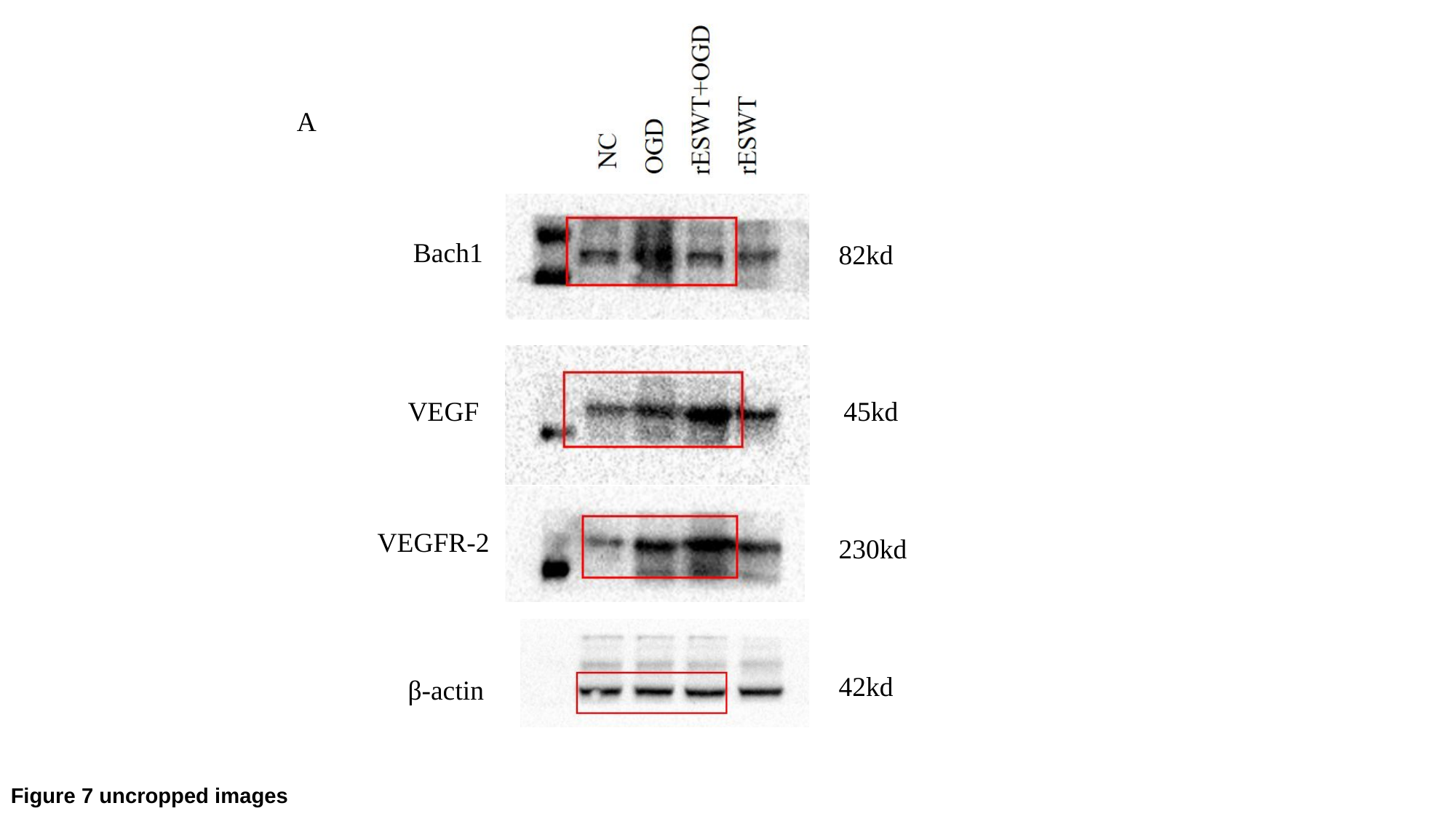

A
Bach1
82kd
VEGF
45kd
VEGFR-2
230kd
42kd
β-actin
Figure 7 uncropped images
